# Crafting inclusive parenting programs– considerations for deaf families: a qualitative study

**DOI:** 10.1186/s13034-024-00852-7

**Published:** 2024-12-19

**Authors:** Asher Emmanuel Ikwara, Rebecca Nabagesera, Isaac Isiko

**Affiliations:** 1 Child Health and Development Centre, School of Medicine,College of Health Sciences, Makerere University , Kampala, Uganda; 2 School of Medicine, College of Health Sciences, Makerere University, Kampala, Uganda; 3 Department of Community Medicine, Axel Pries Institute of Public Health and Biomedical Sciences, Nims University, Jaipur, Rajasthan India; 4 Uganda Virus Research Insititute/UVRI, Entebbe, Uganda

**Keywords:** Deaf families, Inclusive parenting programs, Sign language education, Technological innovations

## Abstract

**Background:**

The parenting of children by deaf parents has many challenges that require a barrier-breaking approach to ensure inclusivity and accessibility. Therefore, this study explored pathways for crafting inclusive parenting programs, fostering a future where every family thrives, regardless of hearing ability.

**Methods:**

This was a qualitative study that employed in-depth interviews with 20 deaf parents and utilized thematic content analysis. This study was carried out at events organized by the Uganda National Association of the Deaf (UNAD) at Makerere College School. Deaf parents were purposively selected from attendees at events organised by the Uganda National Association of the Deaf (UNAD) during the specified celebrations.

**Results:**

An analysis of interviews with deaf parents identified six crucial themes for inclusive parenting programs. Language education was highlighted for strengthening family bonds. Communication and relationship building emphasized equal treatment and love for deaf children. Cultural sensitivity and education were deemed essential, as visual tools and district associations were used. Tailoring content for deaf children emphasized playful and educational materials, such as sign language videos. The utilization of technology for accessible programs and the promotion of community involvement are recognized as critical components.

**Conclusion:**

This study emphasized the unique experiences of deaf parents, highlighting the need for sign language education, cultural sensitivity, and technology while addressing challenges like limited resources, stigma, and advocacy gaps in parenting programs.

## Introduction

In the tapestry of family life, effective parenting programs serve as looms that weave understanding, support, and guidance [1]. However, for deaf parents and their children, the conventional fabric of parenting often involves unique challenges, demanding a nuanced approach to ensure inclusivity and accessibility [2]. This study, titled *“Crafting Inclusive Parenting Programs– Considerations for Deaf Families: A qualitative study”* embarks on a journey to unravel the intricate layers of parenting, focusing on the pivotal aspects that render programs accessible and inclusive for this vibrant demographic.

Deafness is not a barrier to parenting but rather an invitation to reimagine the pathways through which families connect, communicate, and thrive [3]. The overarching aim of this research is to explore and address the unique challenges faced by deaf parents while also identifying pathways to the development of inclusive parenting programs. By exploring the intricate interplay between tailored educational content, community engagement, and the leveraging of technology, this study seeks to identify the essential components that pave the way for inclusive parenting programs.

The World Health Organization (WHO) has provided estimates indicating that approximately 466 million individuals worldwide are experiencing disabling hearing loss, constituting approximately 6.1% of the global population [4]. Within this demographic, 34 million are children, with a predominant concentration in low- and middle-income countries of which Uganda is among. The WHO data shows that the prevalence of hearing impairment, defined as hearing loss greater than 35 dB, among adults aged over 15 years was 15.7% in sub-Saharan Africa, whereas it was 4.9% in high-income countries [5]. In children aged between 5 and 14 years, the prevalence was estimated to be 1.9% in sub-Saharan Africa [6].

Our exploration begins with an examination of the cornerstone of communication for deaf families—sign language education. Within these pages, we delve into the profound impact of equipping parents with linguistic tools to bridge the gap between the hearing world and the deaf world. As a dynamic and expressive mode of communication, sign language not only fosters understanding but also serves as a cultural bridge, anchoring the familial ties that form the heart of effective parenting.

As we navigate through the digital landscape, we also scrutinize the role of technological innovations in rendering parenting programs more accessible for deaf families. The advent of interactive videos, online resources, and communication technologies presents an unprecedented opportunity to create tailored content that resonates with the unique needs of the deaf community. This research examines how such innovations can empower parents, providing them with the resources and support required to navigate the challenges and joys of raising a deaf child.

Furthermore, the manuscript explores the significance of community engagement and awareness campaigns in fostering a culture of inclusivity. By sensitizing the wider community to the rights and needs of deaf children, we aim to break down stereotypes and build a foundation of support that extends beyond the immediate family unit. This study therefore aimed to explore and address the unique challenges faced by deaf parents while also identifying pathways to the development of inclusive parenting programs.

## Methods

### Study setting and study participants

This study was conducted at events organized by the Uganda National Association of the Deaf (UNAD) at Makerere College School, a mixed, public government high school for both Ordinary and Advanced Level students. The school is located near Makerere University School of Law and the College of Education and External Studies along Makerere Hill Road, Kampala, Uganda. Uganda, with an estimated deaf population of approximately 1.3 million people, is home to a significant number of individuals who communicate using Uganda Sign Language (USL), with around 300,000 people utilizing USL as their primary mode of communication [7].

The study involved 20 deaf parents who were purposively selected from attendees at events organized by UNAD during the specified celebrations. These participants were drawn from different regions of Uganda, providing a diverse representation of the country’s socio-cultural context. This geographic diversity helped ensure a broad range of experiences and perspectives, enriching the study’s findings and enhancing the understanding of the challenges and opportunities faced by deaf parents across various parts of Uganda.

### Study design

This research adopted a qualitative approach to investigate the transformative potential of specialised parenting programs for deaf families. The study utilises participatory methods, conducting interviews during the International Deaf Awareness Week and International Day of Sign Language celebrations held from 18th to 22nd September 2023, providing a comprehensive exploration of the experiences and perspectives of deaf parents.

### Eligibility criteria

Eligible participants were parents or caregivers of children with hearing impairment who were willing to provide explicit consent. The inclusion criteria emphasized gender diversity to ensure a balanced representation of perspectives. The exclusion criteria applied to individuals with medical, physical, or cognitive conditions that could hinder their ability to fully engage in the interview process. This includes individuals with severe hearing loss beyond the scope of the study, as well as those with cognitive impairments, mobility issues, or other conditions that would prevent meaningful participation. It is important to note that all participants met the eligibility criteria and were able to contribute meaningfully to the study.

### Data collection and management

In-depth interviews, a qualitative data collection method, were employed to create an interactive and engaging environment for participants. These interviews were conducted by a team of trained researchers who possessed a deep understanding of deaf culture and sign language, ensuring that participants felt comfortable and understood throughout the process. To foster an atmosphere of open and honest communication, the interviews took place in quiet, comfortable spaces that were conducive to effective interaction.

The semi-structured interview format was chosen for its flexibility, allowing the researchers to explore emerging themes while providing a private and safe platform for participants to share their personal experiences. The interview guide was developed with a combination of pre-set questions designed to be open-ended, ensuring that participants could respond in their own words. The guide also allowed for flexibility based on participants’ responses, enabling the researchers to adjust the questions as needed to further probe areas of interest or emerging topics. This iterative approach helped ensure that the interviews remained responsive to the insights and perspectives shared by the participants.

After conducting the initial interviews, the research team began the process of thematic content analysis. The interviews and audio recordings were securely stored, numbered, and transcribed verbatim to ensure accuracy and confidentiality. NVIVO version 12 software was then used to facilitate the systematic analysis of the data. Multiple readings of the transcripts were conducted by the research team to familiarize themselves with the data and ensure thorough understanding. A comprehensive codebook was developed, and iterative coding was performed to identify emerging themes and subthemes. During this phase, the interview guide was refined, with new insights integrated into the coding process to ensure that all relevant themes were captured. The interpretative process involved descriptive analysis, supported by verbatim quotes, to provide rich and meaningful representations of participants’ experiences.

Table 1 below shows the key themes and subthemes that emerged during the analysis, illustrating the depth and breadth of the data.

**Table 1:** Thematic analysis of main codes and sub-codes

Main code/primary code	Sub-code/sub-theme	Sample quote
Importance of sign language education	1. Sign language as a bridge for family communication	“…Sign language has been crucial in helping us communicate as a family; it’s the only way we truly understand each other.”
	2. Strengthening familial bonds	“…It brings us closer, knowing we can express everything, from emotions to everyday needs, through sign language.”
	3. Deeper understanding of deaf children	“…By learning sign language, I understand my child better; it’s not just communication, it’s about connecting with their world.”
Communication and relationship building	1. Paramount role in daily experiences as deaf parents	“Without effective communication, it would be impossible to meet my child’s needs. It is the foundation of our daily interactions.”
	2. Emphasis on care, love, and equality in parental relationships	“Love is not about hearing or not hearing. It’s about how we communicate and care for each other equally.”
Cultural sensitivity and education	1. Prioritizing cultural sensitivity in parenting programs	“Parenting programs must understand our culture; we need approaches that respect our way of life and how we communicate.”
	2. Advocacy for visual tools like sign language skits	“Visual tools like sign language skits are essential; they help not just us as parents, but also those who need to understand our culture.”
Tailoring content for deaf children	1. Need for playful and educative materials addressing deaf culture	“Materials should be fun and educational, incorporating deaf culture to engage our children in a way that speaks to them.”
	2. Importance of videos in sign language for parenting guidance	“I find videos in sign language incredibly helpful, as they give me real-time examples of how to handle parenting situations with my child.”
Utilizing technology for accessible programs	1. Potential of technology in enhancing accessibility	“Technology has opened doors for better learning, making it possible to access content anytime, in our own sign language.”
	2. Creation of videos in the deaf community’s own sign language	“It’s important that videos are made in our own sign language; it helps us connect and understand better than if it’s done in a different sign language.”
Community involvement and sensitization	1. Training parents in sign language	“Training other parents in sign language has been a game-changer; it brings the community closer and helps us communicate.”
	2. Raising awareness about the rights of deaf children	“We need to raise awareness about our children’s rights. Sign language and inclusive programs are crucial to ensuring they receive fair treatment in society.”

#### Reflexivity

Researchers acknowledge their positions and biases, fostering reflexivity in data interpretation for a nuanced understanding of participants’ experiences. The study upheld ethical standards and robust qualitative methodologies to ensure the integrity and reliability of the findings.

## Results

### Demographic characteristics of the participants

The study included a group of 20 participants, consisting of 11 males and 9 females, with ages ranging from 30 to 43 years. The average age of the participants was approximately 37 years, reflecting a broad spectrum of life experiences, as shown in Appendix 1 below. While the gender distribution provides a balanced representation of male and female perspectives within the context of deaf parenting, it is important to note that the sample was not intended to represent a fully diverse range of demographic characteristics. Instead, the focus was on ensuring a balance of gender perspectives to enrich the insights into the experiences of deaf parents.The analysis of interviews conducted with deaf parents revealed six prominent themes, each highlighting crucial aspects of crafting inclusive parenting programs for deaf families as shown in Table 1 below.

### Theme 1

#### Importance of sign language education

Participants unanimously demonstrated the paramount importance of sign language education. Learning sign language was deemed the cornerstone for effective communication within the family unit. Parents expressed that the ability to acquire sign language skills strengthened familial bonds and facilitated a deeper understanding of their deaf children, as illustrated below by some of their statements.

Sign language education is crucial; it serves as the bridge that connects us within our family. As a parent, I witness firsthand how learning sign language enhances our communication, fostering a stronger bond and a more profound connection with my deaf child. (Male, 33 years)

Currently, understanding sign language is like unlocking a door to a richer, more meaningful relationship with my family. It is not just a skill; it is the key to truly comprehending and being understood by my deaf child, promoting harmony and closeness in our everyday interactions. (Female, 32 years)

Parents today recognize the pivotal role of sign language in family dynamics. As we learn and use sign language, we actively contribute to breaking down communication barriers within our homes, nurturing a deeper understanding that transcends words and enriches the fabric of our familial relationships. (Female, 37 years)

The significance of sign language education echoes loudly in the present moment. Parents, including myself, emphasize its paramount importance, as it lays the foundation for effective communication within our families. It is a language of love and understanding that cultivates a harmonious environment, allowing us to connect with our deaf children on a profound level. (Male, 40 years)

### Theme 2

#### Communication and relationship building

The theme of communication and relationship building emerged as central to the experiences of deaf parents. The need for care, love, and a strong parental relationship was emphasized. The participants highlighted the importance of treating deaf children equally, underscoring the necessity of balanced information dissemination to foster meaningful connections, as shown below.

In our daily lives, communication and relationship building are paramount as deaf parents. We emphasize the need for care and love, recognizing that a strong parental relationship is the cornerstone. Treating our deaf children equally is not just a goal; it is a continuous practice that enhances our family bonds in the present moment. (Female, 38 years)

For us, as deaf parents, communication and relationship building is an ongoing journey. We stress the importance of a loving and supportive environment. Currently, we actively work towards treating our deaf children equally, ensuring that balanced information flows freely and fostering meaningful connections within our family. (Male, 43 years)

Communication and relationship building stand out as vital elements in our daily experiences as deaf parents. As we navigate parenthood, we continually underscore the significance of care and love. In the present, we make a conscious effort to treat our deaf children equally, recognizing that balanced information sharing is key to nurturing strong, meaningful connections. (Male, 39 yeats)

The status quo of our lives as deaf parents is that communication and relationship building take centre stage. We prioritize care and love, understanding that a strong parental relationship is foundational. Treating our deaf children equally is not just an ideal; it is a reality we actively create through balanced information dissemination, fostering authentic and meaningful connections within our family. (Female, 35 years)

### Theme 3

#### Cultural sensitivity and education

Deaf parents stressed the importance of cultural sensitivity and education in parenting programs. Sensitizing families to deafness and deaf culture was seen as a key element in building strong familial bonds. Visual tools, such as skits in sign language, were suggested to enhance understanding, whereas district associations of the deaf were identified as potential mediums for disseminating information, as witnessed below by their statements.

In our present reality as deaf parents, we place a high value on cultural sensitivity and education within parenting programs. We actively stress the importance of sensitizing families to deafness and deaf culture as crucial aspects of building strong familial bonds. Today, we advocate for visual tools such as sign language skits, ensuring a deeper understanding and connection within our communities. (Male, 38 years)

Cultural sensitivity and education are ongoing priorities for us, deaf parents. Currently, we emphasize the importance of integrating these elements into parenting programs. Sensitizing families to deafness and the rich deaf culture is more than just an idea; it is a practice we actively promote for stronger familial bonds. Visual tools, such as sign language skits, play a vital role in enhancing understanding within our community. (Female, 35 years)

As deaf parents, we continue to stress the importance of cultural sensitivity and education in the present moment. It is not just about parenting; it is about building a foundation of understanding. Sensitizing families to deafness and our unique culture is a daily goal, and we actively suggest using visual tools such as sign language skits to enrich the learning experience, fostering stronger familial bonds. (Male, 30 years)

In our lives as deaf parents today, cultural sensitivity and education are central themes in our parenting journey. We actively communicate the importance of sensitizing families to deafness and our cultural nuances. Visual tools, particularly sign language skits, are suggested in the present tense as effective means to enhance understanding. District associations of the deaf emerge as key mediums for disseminating this crucial information within our communities. (Male, 36 years)

### Theme 4

#### Tailoring content for deaf children

The need to tailor content for deaf children was a recurrent theme. The participants emphasized the importance of playful and educative materials that address deaf culture and provide guidance on sensitive topics such as adolescence, behaviors, and sex education. Creating videos in sign language was identified as a valuable method for delivering educational content about parenting, as emphasized by the participants below.

… The recurring concern for us as parents of deaf children is the imperative need to tailor content specifically for them. We stress the importance of playful and educative materials that not only embrace deaf culture but also navigate sensitive topics such as adolescence, behaviours, and sex education. Today, we actively advocate for the creation of videos in sign language, ensuring that our children have equal access to vital parenting guidance. (Male, 33 years)

Tailoring content for deaf children is an ongoing focus in our parenting journey. We highlight the importance of incorporating playful and educative materials that address deaf culture and delicately address topics such as adolescence and sex education. Creating videos in sign language becomes a valuable tool, ensuring that educational content is accessible, relatable, and relevant for our children. (Male, 38 years)

As parents of deaf children, the need to tailor content for our kids is a daily consideration. In the present study, we highlight the importance of materials that seamlessly integrate deaf culture while providing essential guidance on sensitive topics such as adolescence and sex education. Today, we actively support the creation of videos in sign language as a powerful means to deliver educational content tailored to the unique needs of our children. (Female, 37 years)

Tailoring content for deaf children is a crucial aspect of our parenting approach, especially in the present moment. We consistently emphasize the importance of playful and educative materials that address our children’s specific needs, covering aspects such as deaf culture, adolescence, behaviors, and sex education. The creation of videos in sign language stands out as a valuable method, ensuring that the content is not only informative but also accessible and engaging for our deaf children. (Female, 40 years)

### Theme 5

#### Utilizing technology for accessible programs

Participants recognized the potential of technology in enhancing accessibility to parenting programs. The creation of videos in sign language and the utilization of online resources were highlighted. The importance of embracing technology in the deaf community’s own sign language, rather than relying solely on content in American Sign Language, is underscored below.

In our present reality, the potential of technology in making parenting programs accessible for all is undeniable. We actively advocate for the creation of videos in our own sign language, ensuring that the content resonates with the deaf community. Embracing technology means more than just online resources; it is about having content that speaks our language and caters to the diverse linguistic needs within our community. (Female, 39 years)

As parents, we are currently witnessing the transformative power of technology in accessing parenting programs. In the present study, we emphasize the creation of videos in our unique sign language to ensure inclusivity. It is not just about online resources; it is about embracing technology that speaks directly to us, allowing us to participate fully in the wealth of parenting knowledge available. (Male, 41 years)

The present moment highlights the incredible potential of technology in our parenting journey. We actively promote the creation of videos in our own sign language, recognizing the importance of tailored content for the deaf community. Embracing technology extends beyond mere accessibility; it is about having programs that genuinely resonate with us and cater to the linguistic diversity within our community. (Male, 39 years)

Today, the deaf community sees first-hand the impact of technology on accessible parenting programs. Additionally, we emphasize the creation of videos in our own sign language as a crucial step toward true inclusivity. Embracing technology means going beyond generic online resources; it is about having content that reflects our linguistic identity, ensuring that all deaf individuals can benefit from the wealth of parenting information available. (Female, 42 years)

### Theme 6

#### Community Involvement and Sensitization

Community involvement and sensitization emerged as critical components of inclusive parenting programs. Training parents in sign language and disseminating awareness through various media were emphasized. The participants stressed that raising awareness of the rights of deaf children is foundational, contributing to an environment where inclusivity naturally falls into place, as shown below.

…Community involvement and sensitization stand as pillars in our commitment to inclusive parenting programs. We actively engage in training parents in sign language, fostering a community that embraces communication diversity. Raising awareness about the rights of deaf children is not just a goal; it is a daily practice that contributes to an environment where inclusivity becomes a natural and integral part of our shared experiences… (Female, 42 years)

For us, community involvement and sensitization are ongoing endeavors in our parenting journey. We prioritize training parents in sign language, recognizing its role in creating a more inclusive environment. Raising awareness about the rights of deaf children is a continuous effort, contributing to a community where inclusivity is not just a concept but a lived reality. Female, 35 years)

Community involvement and sensitization take center stage in our lives as parents… Actively training parents in sign language is a fundamental step toward inclusivity. We consistently advocate for raising awareness about the rights of deaf children, understanding that this proactive approach fosters an environment where inclusivity is not only encouraged but ingrained in our community fabric. (Male, 41 years)

Today, community involvement and sensitization are integral aspects of our commitment to inclusive parenting. Currently, training parents in sign language is a proactive step toward building an inclusive community. Raising awareness about the rights of deaf children is a daily focus, laying the groundwork for an environment where inclusivity is not just a concept but a shared value within our community. (Male, 36 years)

In summary, these themes collectively underscore the multifaceted nature of inclusive parenting for deaf families, emphasizing the integral role of sign language education, effective communication, cultural sensitivity, tailored content, technology, and community involvement. The findings of this study provide valuable insights for the development of parenting programs that break barriers and foster inclusivity for deaf parents and their children.

## Discussion

This study findings illustrated insightful experiences and needs of deaf parents, particularly in relationship to the study topic on the development of inclusive parenting programs. From the analysis, six key themes were identified and they included;—*Importance of Sign Language Education*, *Communication and Relationship Building*, *Cultural Sensitivity and Education*, *Tailoring Content for Deaf Children*, *Utilizing Technology for Accessible Programs*, and *Community Involvement and Sensitization*—and this we believe were able to highlight essential elements that must be considered when designing effective parenting programs for deaf families.

Importance of Sign Language Education was identified as foundational for communication within deaf families. The findings reinforce the idea that sign language plays a pivotal role in building strong familial bonds and facilitating deeper emotional connections between deaf parents and their children. However, participants also emphasized the challenges they face in accessing high-quality sign language education, particularly with the lack of affordable or accessible language classes and teachers fluent in specific dialects. This gap in resources creates a barrier that can limit both the emotional development of the parents and the overall family dynamic.

Communication and Relationship Building emerged as another essential element in the parenting experience, highlighting the importance of emotional care, love, and equality in family relationships. Although sign language was central to communication, many parents voiced concerns about reaching full emotional and developmental engagement with their children, especially in areas such as adolescence, sexuality, and education. Societal attitudes towards deaf families were also cited as an obstacle, with persistent stigma and misconceptions hindering the development of inclusive, supportive environments. This shows the ongoing challenge of overcoming societal barriers to effective deaf parenting.

Cultural Sensitivity and Education emerged as another key theme. Participants stressed the need for culturally sensitive parenting programs that incorporate deaf culture into their curriculum to ensure inclusivity and understanding. However, many participants expressed frustration with the limited availability of culturally relevant parenting materials. Deaf organizations at the district level provided some resources, but these were often outdated, inadequate, or difficult for families in rural or underserved areas to access. The lack of accessible, culturally appropriate education, particularly in mainstream programs, represents a significant gap that needs addressing.

Tailoring Content for Deaf Children pointed to the need for educational materials that cater to the unique needs of deaf children. Many parents felt that existing resources were not sufficiently designed for deaf children, often lacking playful, engaging content or failing to address sensitive topics like adolescence and sex education in culturally relevant and accessible ways. The scarcity of tailored resources, particularly those accommodating different sign languages, adds another layer of complexity to providing effective educational and emotional support to deaf children.

Utilizing Technology for Accessible Programs showed the potential for digital platforms to enhance accessibility for deaf families [8]. Sign language videos and online resources were highly praised for their effectiveness in ensuring parenting programs are inclusive and cater to the linguistic needs of deaf families. However, accessibility to these resources is not universal. Families in rural or low-income areas may not have reliable access to the internet or modern devices, highlighting the digital divide as a significant barrier to ensuring all deaf families can benefit from technology-enhanced programs.

Community Involvement and Sensitization focused on the role of the community in supporting deaf families. Participants emphasized the importance of training hearing parents in sign language and raising awareness about the rights of deaf children. A common theme throughout the study was the lack of adequate advocacy and support from local governments or community organizations. Despite their willingness to engage in advocacy efforts, many deaf parents lack external assistance in navigating their parenting journey, including securing necessary educational and social inclusion for their children.

The findings of this study align with and extend previous research on deaf parenting and inclusive education. Research by [9] and [10] emphasizes the centrality of sign language in fostering communication and emotional bonds between deaf parents and their children, which this study supports [11]. However, this research also highlights the challenge of accessing sign language education, particularly in rural areas, which was less frequently addressed in earlier studies.

Similarly, the findings echo those of [12], who found that deaf parents who use sign language have stronger emotional connections with their children. However, this study adds nuance by emphasizing the difficulties these parents face in consistently accessing sign language resources.

In contrast, the study diverges from earlier works such as [13] by not only emphasizing the role of sign language but also calling for culturally sensitive parenting programs. Dammeyer’s research focused more on sign language but did not address the integration of deaf culture into parenting programs, a gap that this study identifies as crucial for fostering inclusive parenting environments.

The study’s exploration of technology aligns with [14], who examined the role of digital platforms in enhancing accessibility for deaf parents. However, this study goes a step further by acknowledging that digital resources are not universally accessible, highlighting the need for equitable access to these technological tools.

This study contributes to the theoretical framework surrounding inclusive parenting by incorporating cultural sensitivity and technology—two elements less emphasized in previous models. It advocates for a comprehensive approach to deaf parenting that addresses communication, cultural understanding, and technological accessibility.

Practitioners should prioritize integrating sign language education and culturally relevant content in parenting programs. They must also acknowledge barriers to access, such as socio-economic status, rural location, and limited digital access, and work toward closing these gaps to ensure that all families can benefit from these programs.

Policymakers should use the findings of this study to inform public health initiatives, educational programs, and social services which aligns with the study by [15]. There is a pressing need for policies that promote the development of culturally tailored resources for deaf families, support the widespread usage of sign language in community-based programs, and address access issues related to digital resources.

Theoretically, this study advances our understanding of inclusive parenting by integrating sign language, cultural sensitivity, and technology into a comprehensive model. Practically, it offers actionable insights for the design of parenting programs that cater to the unique needs of deaf families, ensuring that they have access to the necessary resources and support to thrive as parents. While this study provides valuable insights, it is important to consider alternative interpretations. For instance, the emphasis on sign language may overlook other communication strategies used by some deaf families, such as lip-reading or written communication. Further research could explore a wider range of communication strategies to provide a more comprehensive view of deaf parenting. Additionally, while technology is a powerful tool, it is essential to address access disparities to ensure that all deaf families can benefit from digital resources.

### Strengths and limitations

This study’s strengths lie in its qualitative approach, which allowed for an in-depth exploration of the lived experiences of deaf parents. Semi-structured interviews provided nuanced data, capturing diverse perspectives and revealing both strengths and challenges in the parenting journey of deaf families.

However, the study also has limitations. The sample size (20 participants) may not fully represent the broader population of deaf parents, and the sample was predominantly composed of individuals from similar socio-economic backgrounds, which may not capture the full diversity of experiences. Future research should aim for a more diverse sample, including participants from varied socio-economic and geographical backgrounds, to broaden the scope of understanding.

### Future research directions

Future research could explore the perspectives of other stakeholders—such as educators, policymakers, and service providers—to gain a more holistic understanding of how inclusive parenting programs can be developed and implemented. Longitudinal studies could assess the long-term impact of culturally sensitive and technology-enhanced parenting programs on the well-being of deaf families. Additionally, exploring the barriers deaf parents face in accessing existing parenting resources, such as financial constraints, limited availability of sign language classes, or social stigma, could offer valuable insights for the development of more inclusive services.

## Conclusion

This study contributes to the unique experiences of deaf parents and underscores the importance of sign language education, cultural sensitivity, and technology in developing inclusive parenting programs. While these factors present opportunities for improving the support offered to deaf families, the study also highlights significant challenges, including limited access to resources, societal stigma, and insufficient advocacy. These findings contribute to the growing body of knowledge on inclusive parenting and provide actionable recommendations for designing programs that address both the opportunities and barriers faced by deaf families.

### Recommendations

In the amalgamation of these diverse insights, this study propels us toward the development of inclusive parenting programs that transcend barriers and cultivate an environment conducive to the optimal development of deaf children. By integrating sign language education, emphasizing communication, promoting cultural sensitivity, tailoring content, utilizing technology, and encouraging community involvement, these programs hold the promise of breaking barriers and fostering inclusivity for deaf families.

## Appendix 1: Showing the demographic characteristics of the participants

**Table:** 

Participant	Gender	Age
P1	Male	33
P2	Female	32
P3	Female	37
P4	Male	40
P5	Female	38
P6	Male	43
P7	Male	39
P8	Female	35
P9	Male	38
P10	Female	35
P11	Male	30
P12	Male	36
P13	Male	33
P14	male	38
P15	Female	37
P16	Female	40
P17	Female	39
P18	Male	41
P19	male	39
P20	Female	42

## Data Availability

The raw data supporting the conclusions of this article will be made available upon request from the corresponding author.

## Abbreviations

IDAW: International deaf awareness week
IDSL: International day of sign language
WHO: World health organization
UNAD: Uganda national association of the deaf
